# Pragmatic trials and implementation science: grounds for divorce?

**DOI:** 10.1186/s12874-019-0814-9

**Published:** 2019-08-16

**Authors:** Ray Pawson

**Affiliations:** School of Sociology and Social Policy, University of Leeds, Leeds, LS2 9JT UK

**Keywords:** Pragmatic trials, Implementation science, PRECIS models, Generalisability, Research users, Multi-methods, Heterogeneity of treatment effects, Within-case and cross-case analysis

## Abstract

**Background:**

The paper opens with a brief history of two of the major intellectual components of the recent utilitarian turn in clinical research, namely **‘**pragmatic trials’ and ‘implementation science’. The two schools of thought developed independently and the paper scrutinises their mutual compatibilities and incompatibilities, asking: i) what do the leading advocates of pragmatic trials assume about the transfer of research findings to real-world practice and ii) what role pragmatic trials can and should play in the evaluation of implementation science strategies.

**Methods:**

The paper utilises ‘explication de texte’: i) providing a close reading of the inferential logics contained in major published expositions of the two paradigms, and ii) interrogating the conclusions of a pragmatic trial of an intervention providing guidelines on retinal screening aimed at family practitioners.

**Results:**

The paper is in two parts. Part 1 unearths some significant incommensurability – the pragmatic trial literature retains an antiquated view of knowledge transfer and is overly optimistic about the wide applicability the findings of pragmatic trials to ‘real world’ conditions. Part 2 of the paper outlines an empirical strategy to better penetrate the mechanisms of knowledge transfer and to tackle the issue of the generalisabilty of research findings in implementation science.

**Conclusions:**

Pragmatism, classically, is about problem solving and the melding of perspectives. The core research requirement in implementation science is a fundamental shift from the narrow shoulders of pragmatic trials to a model of explanation building based upon a multi-case, multi-method body of evidence.

## Background

The previous decades have seen a marked utilitarian turn in clinical research and the paper compares and contrasts two of its major intellectual components. In 1967, Schwartz and Lellouch founded the idea of a ‘pragmatic attitude’ in trial methodology [1]. Instead of explanatory (or efficacy) trials conducted with a large battery of premeditative controls, they promoted the introduction of pragmatic trials operating in and thus more reflective of the hurly-burly of real world treatments. In 2006, Eccles and Mittman welcomed us to ‘implementation science’ with the same emphasis on improving routine care, in this instance by promoting and devising methods which promote the systematic uptake of research into practice [2]. Both ideas have created enormous literatures, both engage with programme complexity and both are said to have broadened the academic mindset [3]. There is a case declaring a marriage made in methodological heaven [4].

Despite their common ambitions this contribution unearths significant incompatibilities between the two schools of thought. Part 1 of the paper provides a brief and critical history of each perspective revealing some latent tensions on the signature issues of research utilisation and the generalisabilty of research findings. More specifically it is argued, despite many claims to the contrary, that pragmatic trials have no claim to widespread applicability. Accordingly, when they are put to use in evaluating knowledge transfer schemes, the findings are case specific and not generic as often supposed. This thesis is supported by examining the respective inferential logics of the two paradigms and then with an illustration of a pragmatic trial on the utility of ‘educational reminders’ to family/general practitioners.

As with many marriages some counselling and support may be in order. The role of pragmatic trials needs careful re-specification. Part 2 of the paper outlines a strategy that may be more useful in garnering the generic lessons of implementation science. The core requirement is for a method that can explain outcome heterogeneity and for this we need research designs that are theory-driven and tested using a multi-case, multi-method body of evidence. This model is illustrated using the same example.

## PART 1: grounds for divorce

This section provides basic background material on the two schools of thought. They are then placed eyeball to eyeball. What do pragmatic trials assume about research utilisation? What does implementation science make of pragmatic trials?

### Pragmatic trials

Let us begin with a simple description of the original motivation behind the shift to pragmatic trials. The classic RCT – the explanatory or efficacy trail – employs random assignment, blinding, and then further controls for selection bias, performance bias, adherence bias, detection bias, attrition bias, reporting bias and so on [5]. This design maximises the chances of uncovering a positive effect in favour of the treatment and is said to permit the inference that the intervention and the intervention alone has causal efficacy. Such a strategy, however, is considered a poor guide to real-world effectiveness where such controls do not apply and treatment is applied to patients: with co-morbidities and uneven compliance propensities; by practitioners with different experience and preferences; with the disparate stakeholders seeking different, short and long-term outcomes; and so on. The solution, the clarion call, is clear – design pragmatic trials (PRCTs) that are: i) more responsive to the real-world conditions and ii) more informative to real-world practitioners.

In the early literature, these two benefits were pounced upon with considerable relish. Dozens of authors used the ‘real-world’ motif to describe the target domain of a pragmatic trial. Flay el al, for instance, used the following definition: ‘A test of whether a program does more harm than good when delivered under real-world conditions’ [6]. Karanicolas et al. provide this stipulation: ‘A trial is practical to the extent that it provides comprehensive information that bears on real-world healthcare decisions’ [7]. Patsopoulos, in a much cited paper, offers: ‘The research question under investigation is whether an intervention works in real-life’ [8]. By moving the trial from ‘ideal’ to ‘real’ world conditions, the initial expectation was that a study’s findings could be generalized to other (unspecified and more general) samples and settings. Ware and Hamel [9], for instance, make the following bold declaration: ‘Pragmatic trials are designed to study real-world practice and therefore represent less-perfect experiments than efficacy trials: they sacrifice internal validity to achieve generalizability’.

As well as the claim about the real-world settings of PRCTs, the early literature goes on to stipulate much more about the beneficiaries of the evidence obtained from trials conducted in everyday locations. Schwartz and Lellouch instigated this theme differentiating the explanatory aim of ‘acquiring information’ from the pragmatic goal of ‘making a decision’ [1]. Other contributions go on to identify the decision-makers. Wasan considers that pragmatic trials are there to help ‘providers’ [10]. Other accounts begin to multiply the stakeholders, such as Flay’s ‘prescribers and health care planners’ [6] as well as Ware and Hammel’s ‘patients, clinicians and policy makers’ [9]. The contribution that pushes hardest for a user or audience-based interpretation of the aims of pragmatic trials is that of Tunis el al, who list potential recipients as follows: ‘clinical and health decision makers including patients, physicians, payers, purchasers, health care administrators and public health policy-makers’ [11].

These twin aspirations have generated considerable excitement not to say paradigm change in the world of clinical trials. Patsopoulos [8] monitors a hundredfold increase in MEDLINE abstracts from 1990 to 2010, which use the terms ‘pragmatic’ or ‘naturalistic’ to describe the reported RCT. Over the same period the technical apparatus for differentiating pragmatic trials from explanatory trials became much more exacting. These methodological exercises set themselves the task of stipulating the precise dimensions on which the two approaches differed. What exactly would the explanatory trial seek to control and the pragmatic trial permit to vary? A range of formalisations, typologies and models have been devised for the purpose.

The best known typology is probably that of Sackett, which describes nine different components upon which trials may differ: participant eligibility, experimental intervention, comparison intervention, practitioner expertise, participant compliance with intervention, practitioner adherence to protocol, follow-up intensity, primary outcome and primary analysis [12]. The technical content of each component is closely defined as are the extreme poles of each dimension. On each of these dimensions a trial may be relatively ‘explanatory’ or ‘pragmatic’ with the latter having more potential to inform routine treatment.

**Fig. 1:**
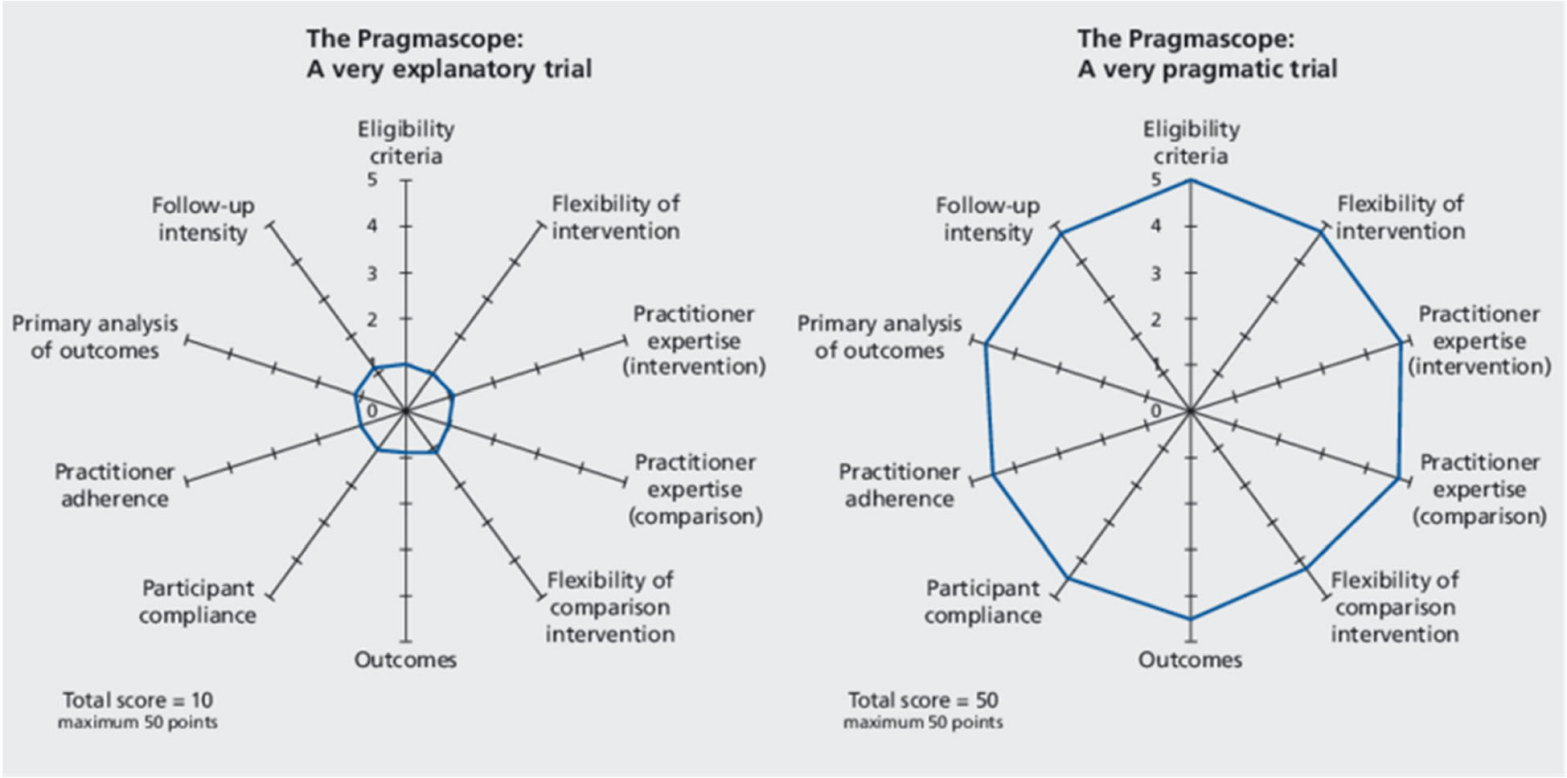
The pragmascope. Source [15] The *Journal of the American Board of Family Medicine*

The best known model is, of course, the PRECIS tool (PRagmatic Explanatory Continuum Indicator Summary) with its famous spokes-hub-and-rim, bicycle-wheel diagrams [13, 14]. The current PRECIS-2 tool also defines 9 dimensions (the spokes) with which to differentiate explanatory and pragmatic trials. Researchers assess any present or proposed trial by applying a score (Likert-scale 1–5) on each dimension, with more explanatory designs being placed near the hub and more pragmatic trials located nearer the rim. The overall configuration (relatively explanatory or relatively pragmatic) is then signified by a perambulatory line which connects up the selected scores. Figure 1 provides an idealised illustration, taken from an early version of PRECIS, carrying the splendid title – the ‘pragmascope’ [15]. The two figures contrast highly pragmatic and highly explanatory RCTs. All real applications of the tool uncover significant fluctuations between hub and rim as they traverse the spokes (e.g. [16]).

The PRECIS wheels have proved an eye-opener. The complexity of the trial apparatus is laid bare. Those tricky design choices on patient groups, practitioner expertise, comparison groups, outcome measures, follow-up periods and so on, which often remain submerged in trial publications, are now made explicit. But something else also becomes perfectly clear, namely that *other* PRCTs on the *same* treatment could well configure the trial in multiply different ways. For perfectly good practical reasons another trial might prefer an wider patient group, an alternative set of practitioners, another usual-care comparison group, a proxy outcome, a shorter follow-up period, and so on. Such is pragmatism. And what crumbles on this realisation is the original ambition that pragmatic trials have wider applicability, namely that they are able to speak to the generality of real-world conditions. The authors of PRECIS-2 make this reversal perfectly transparent: ‘Our conception of PRECIS-2 is that it is to be used by trialists to design a trial whose results are applicable to a context in which they, the trialists, are intending the results to be used’ [17]. In short, the revised model of research utilisation is to extrapolate findings from a specific pragmatic trial to a specific application in which the same operational and contextual conditions apply.

There has been a similar volte-face when we come to post-PRECIS ambitions on who will use the findings from pragmatic trials. The original expectation was wide-eyed – recall Tunis et al’s formidable roll call of users [11]. But now it is necessary to face the inevitability that different potential users working in different settings might want different information from that provided in a specific PRCT, which has examined one patient group, one set of practitioners, one comparison group, in one time frame, etc. That penny having dropped, the solicitation of practitioners becomes muted. Here are two such examples from the PRECIS team:

‘We would argue that trialist should not worry about trying to guess the various perspectives of those making decisions but should instead do all they can do to describe the context of their trial’ [18].

‘But this judgement of similarity, and thus of applicability, is really a decision about local implementation, and is not the responsibly of the trial design team, but of the reader who may be a decision maker and potential user of the evidence provided in the published RCT’ [17].

With this acknowledgment the expectations on knowledge transfer become formidable. The PRCT researcher must author publications with extensive contextual information – ideally by using the ‘CONSORT extension for Pragmatic Trials’ [19]. The circumstantial detail supplied in the said publication, the argument continues, is thereby rendered sufficiently comprehensive for ‘future readers of that study to judge the applicability of the results to their own context’ [17]. As we are about to see, this writers-and-readers view research implementation is profoundly antiquated.

### Implementation science

This section outlines the basic principles of implementation science. It reverses the previous vantage point, raising two questions: i) does the understanding of research utilisation assumed in the PRECIS tool comply with the frameworks for knowledge transfer as established in implementation science? ii) to what extent and with what success has implementation science used pragmatic trials as a source for evidence for evaluating research utilisation strategies? The answer to the first question is a short and resounding ‘no’ – though I spend a few paragraphs explaining why. The second question is more challenging. Whilst there is considerable usage of pragmatic trials in implementation research, its benefits remain open to debate.

As we have seen, PRCTs in general and PRECIS in particular presume a ‘library model’ of research utilisation. Researchers, under the watchful eye of CONSORT librarians, deposit their papers. Borrowers search the shelves and catalogues to find items that match their particular interests and inform their actions. Is this a viable model? There are approximately a million papers from clinical trials in the library [20] and scant evidence that they have a significant practitioner readership. This evidence comes from a variety of sources. There is a considerable anecdotal literature in the professional journals in which exasperated practitioners explain why they don’t read research – along these lines: ‘too hard pressed to bother with all that statistical gobbledygook’ [21]. More significantly, there is also a mass of formal evidence in the ‘barriers and facilitators’ tradition on why research readership is so low. These studies have been conducted on most professional groups – nursing, primary care, hospital/specialist care, rehabilitation, management and policy makers (for a summary see [22]). The reported barriers are much the same – lack of time, lack of knowledge, lack of access, lack of medical resources, patient-driven priorities, depreciatory attitudes to academic research, financial constraints, etc. Finally, one might consider the negative impact on readership of polemical blasts from clinical insiders who go so far as to declare that ‘most published research findings are false’ [23]. All in all there is little evidence that intrepid practitioners spend time searching for specialised trial evidence and little motivation for them to do so. The same conclusion beckons, this time as acknowledged by Oxman et al.: ‘the primary usefulness of the pragmatic-explanatory framework is for those designing trials, not for users of trials’ [24].

Clearly there is a vacuum to be filled and strategies variously named ‘implementation science’, ‘translational research’ and ‘research utilisation’ have mushroomed in recent year with the goal of devising and promoting strategies to promote the systematic uptake of research into practice. The core ideas are well established: i) knowledge transfer needs to be pro-active rather than reactive, ii) research only becomes useful if it is co-produced rather than dispatched to the ether, iii) accommodating new research ideas requires dynamic, multi-level change involving both individuals and institutions.

A whole array of measures has been put in place to achieve these ends, which include improved targeting, better presentation, using incentives and funding formulas, new e-communication strategies, involving research champions and lay promoters, more patient-centred research, improving practitioner resources, and all combination thereof. For a glossary see [25]. It goes without saying that none of these interventions is a panacea, all of them work to a limited extent in limited circumstances. And implementation science has also busied itself charting the mediators and moderators, the facilitators and barriers, which propel and/or limit success. These models have become more and more compendious over the years, cumulating in a much cited ‘consolidated framework’ [26], which provides an overview of the many requirements that require attention in advancing implementation. Damschroder et al. propose that knowledge transfer requires a complex scaffolding, ‘composed of five major domains: intervention characteristics, outer setting, inner setting, characteristics of the individuals involved, and the process of implementation’. Each of these constructs is then subdivided leaving the KT specialist to manage change on no less than 35 dimensions.

I refrain from the debate about whether such models have become over-elaborate [27], the point being that – even if they are only minimally correct – they demonstrate instantaneously the wishful thinking in the superficial PRECIS model of research implementation. Researchers can be as diligent and comprehensive as they like in their publications. But getting research into practice is a skilled accomplishment requiring a whole network of planned and adaptive contributions, tailor made to local circumstances.

This brings us to our second question about the place of pragmatic trials in implementation science. Just as it is always vital to ask of a treatment ‘does it work’, we are entirely justified in putting the same question to these implementation programmes. There is a clear need to plan bespoke knowledge exchange strategies, devise fresh communication processes, create new translational networks, roles and partnerships, and so on. But do they really do the job of getting research into practice? Can they really inspire the reluctant? These are empirical questions and they require the selection of appropriate evaluation research methods with which to answer them. Justifications have been forwarded for many different primary and secondary techniques as the method of choice for implementation science. Our interest here is in the use of pragmatic trials in evaluating the schemes that have sought to close the research-practice gap.

It turns out that PRCTs find considerable usage in this enterprise. A key word search for ‘pragmatic trials’ in *Implementation Science* delivers 273 results (June 2018). A tiny fraction of these are methodological commentaries, the vast majority use the PRCT to evaluate particular schemes devised to promote and increase research usage. This methodological choice should not come as a surprise. The RCT remains the default setting in most corners of evidence-based medicine – holding to the nostrum that no other study design provides the power to balance known and unknown prognostic factors at baseline. However, in the evaluation of knowledge transfer schemes the opportunity to mount the ‘gold-standard’, efficacy trial is, by common consent, greatly restricted. Subjects cannot be blinded and it usually impossible to randomise the implementation at the level of the individual recipient. Accordingly, cluster randomisation is the norm and this design passes much of the management of the schemes to the responsible healthcare agencies, which introduces inevitable local variation in their implementation. In short, implementation science deals with notoriously ‘leaky’ social interventions, which pass through many hands in many different circumstances, and which are thus considered ripe for evaluation using ‘real world’ pragmatic trials.

### Printed educational messages

Having identified the PCRCT (pragmatic cluster randomised controlled trial) as a significant approach in implementation science, I want to return to the paper’s core concern – do the results of such investigations apply generally or are they case specific? Readers will recall the profound change of heart on intended usage and users described in section 1.1. As one trawls the 250+ papers mentioned above these divergent ambitions become apparent. It is possible, however, to exemplify the dilemma on the basis of a single paper, namely Zwarenstein’s et al’s 2014 study of printed educational messages aimed to increase retinal screening amongst patients with diabetes [28]. The existing evidence base showed that failure to screen for retinopathy exposed primary care patients with diabetes to risk of eye complications. Screening was initiated by referral from family practitioners but adherence to existing guidelines was known and shown to be suboptimal. A programme was devised that sought to boost referrals with the use of ‘reminders’ to physicians in the form of printed educational messages (PEMS).

This brings us to the evaluation, which sought to test the effectiveness of different reminder formats (‘inserts’, ‘outserts’, ‘patient addendums’) included in a mailed newsletter distributed regularly to 15,000 healthcare providers in Ontario. Practices were randomly selected to receive an issue including or omitting the retinopathy guidance. The outcome measure compared the number of referrals in a given period across the various experimental and the control practices. Such cluster trials are notoriously difficult to mount and to analyse [29] and the present study is chosen because it is exemplary in technical terms (data sources, baseline measures, patient tracking, statistical power, and so on). No intervention effect was detected, with referral rates being remarkably consistent across all arms of the trial [28].

The question is – to whom and in what circumstances does this result apply? Does it speak to the generality of educational guidelines/reminders to family/general practitioners or does it relate only to the specifics of the condition, the personnel, the delivery, the time and the place of this intervention. Readers might like to check this out for themselves; but my interpretation is that Zwarenstein et al’s paper suggests a ‘bit of both’. The trial is introduced in the context of contradictory evidence from reviews of a variety of PEMs directed at a variety of conditions and claims to be able to resolve the inconsistency with ‘large, well-designed RCTs on the effect of PEMs on guideline adherence, conducted in real world settings amongst typical practitioners’ [28]. This image of a representative intervention is reinforced at several points as with the description that the study ‘was conducted under realistic conditions to mimic typical programme delivery’ and was ‘a faithful operationalisation of the sort of printed educational materials that are routinely used’. All leads to a conclusion that conspires cleverly to be *both* narrowly-pitched and far-reaching: ‘This large trial conclusively failed to demonstrate any impact of printed educational messages on screening uptake. Despite their low cost, printed education messages should not be routinely used in attempting to close evidence-practice gaps relating to retinopathy screening’ [28].

What we have here is an echo of the entire history of pragmatic trials, which began with an appetite for widespread applicability but which shrunk subsequently a diet of case-to-case correspondence. As is now conceded, the latter understanding is correct [17]. We can say that these particular results are conditioned by and apply to only to specific retinal screening PEMs delivered to specific practices in Ontario. They speak only to the specifics of this schema – e.g. free and/or insured provision, easy access to highly qualified FPs/GPs, high levels of screening provision, routine mailing systems, good data base linkage, similar patient demographics and so on (recall the consolidated list of implementation characteristics in [26]). Even the turn of history is important; these results reported in 2014 relate to an intervention mounted 2005. How long before PEMs will be replaced by WETs (weekly educational tweets)?

As can be seen in this example, researchers conducting pragmatic trials sustain a perfectly understandable yearning for broad ‘real-world’ application. They want to deliver an evidence-base that is informative beyond the confines of the given experimental set-up. And yet leading methodologists advocating pragmatic trials now avoid all such claims; inferential logic requires that the results of a specific pragmatic trial relate only to other interventions with the same configuration of implementation characteristics and contextual conditions.

Is a there a resolution?

## PART 2: the case for reconciliation

Indeed there is. To risk further torture of the paper’s divorce metaphor I want to suggest that the PRCT should mend its ways, seek mediation, and call on further support. All of the schemes mounted to improve research uptake are indubitably social interventions and in the evaluation of such programmes there is no agreed ‘hierarchy of evidence’ but rather a ‘bricolage of approaches’. Implementation science, perhaps more than any other healthcare domain, has striven for and argued over the optimal approach to such mixed-method or hybrid designs. I avoid any attempt to win the paradigm wars here. Part 2 takes on a simpler task, namely to suggest using the opportunity presented by PRCTs in a different, more inclusive way. It begins by outlining a research strategy for learning the generalisable lessons of implementation science (Table 1) and concludes with a brief example exemplifying the approach.

**Table 1:** Evaluating generic implementation strategies

*Step one.* Regard randomised trials as ‘case studies’ evaluating just one out of a kaleidoscope of different configurations in which a knowledge transfer scheme may be implemented. Deepen the case study by using multiple methods – examine not only the trial outcomes but also the underlying interpretative processes which may account for intended and unintended outcomes.	
*Step two.* Change the unit of analysis from the programme to the programme theory. Complex interventions are enormously difficult to duplicate but programme theories are readily transportable; the same ideas recur over and again in the world of programme planning. Interrogating these generic programme theories – rather than specific interventions – opens the door to more generalisable findings.	
*Step three*. All knowledge transfer initiatives have mixed fortunes. Change emphasis in outcome analysis from the measurement net effects to the inspection of heterogeneity of treatment effects (HTE), including instances of programme failure.	
*Step four.* Use ‘within-case’ analysis to raise and test hypotheses on why the implementation theory works only for some practitioners. Use ‘cross-case’ analysis to raise and test hypotheses on why the implementation theory works only in some institutional contexts. Repeat indefinitely.	

Source (author)

Put like this, such an agenda can seem horribly abstract. To which I respond in two ways. Firstly, this view of the cumulation of knowledge has a considerable philosophical pedigree [30]. Secondly, the model finds practical usage if one examines the body of research on an intervention rather than individual contributions.

**Fig. 2:**
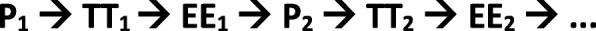
Popper’s Theory of the Growth of Knowledge as ‘Conjectures and Refutations’. Source (author)

The research process suggested in Table 1 corresponds closely to Popper’s theory of the growth of scientific knowledge, which rests heavily on the idea of progressive ‘error elimination’ (Fig. 2). As applied to implementation science, the strategy unfolds as follows. Research begins with the initial identification of a problem (P_1_), in this case about lack of knowledge transfer. A tentative programme theory (TT_1_) is put forward which might offer a solution, as embodied in a particular scheme to improve the uptake of research into practice. The scheme is put to research and invariably meets with mixed success. This finding is regarded as provisional and capable of revision in the light of later findings. Further progress depends on inspecting both the successes and failures of the programme with special emphasis on the elimination of the errors (EE_1_). This scrutiny leads to a more nuanced understanding of the problem (P_2_), a refined solution (TT_2_), and more research, favourable and otherwise. The elimination of further errors (EE_2_) is the spur to progressive knowledge. The process then repeats indefinitely.

Is there a process of conjectures and refutation within implementation science? As noted, many authors are prone to convey an undue sense of certainty, finality and universality in their research findings. But at the same time, if one examines progress across the body of research, a more tentative and truthful tale is to be told. We turn to the literature for an exemplification of Table 1.

### Step one

Urges that PRCTs widen their remit and become multi-method case studies. The advice that RCTs should incorporate a qualitative element is entirely commonplace, though there are differences of opinion on whether the liaison is best served though open-ended interviews, process evaluation, intervening variable analysis, theory-based evaluation, realist evaluation and so forth. Consistent with all of these, if rarely put this way, is the suggestion that such inquiries should also be considered case studies, investigating one of many ways in which such interventions might be configured.

We don’t have to look too far for an example here, for the PEMs study [27] was indeed partnered by a ‘theory-based process evaluation’ [31]. The latter study used a mix of standardised questionnaires and open ended interviews to ascertain physicians’ attitudes towards and experience of the intervention and in so doing it begins to explain the null result of the PRCT. Put briefly, the qualitative study discovers positive attitudes towards retinopathy screening, deep knowledge of its availability, and a strong intention to enact the referrals, which exists before and after the intervention. And yet we know from the PRCT that referrals rates remain static (and disappointing). The explanation emerging from the open-ended interviews is that a range of ‘post-intentional’ factors blunt the espoused willingness to refer. These are presented by way of illustrative quotations from the recipients, which address a diverse bundle of practical concerns – physicians’ preference for their own judgement, contrasting views on the effectiveness of screening, time constraints and pressures, the administrative burden of referral, some patients’ disinterest in screening, non-coverage of screening under some insurance plans, long waiting times and the inaccessibility of some screening provision [31].

We have first sight of a virtuous circle of explanation. The rigorous PRCT secures the outcome finding but cannot say why the intervention is ineffective. The qualitative interrogation delves into a range of underlying processes at work showing how physician experience, patient preferences and administrative constraints may have combined to generate the unanticipated outcome. This constitutes a highly plausible account of programme failure *but one that cannot yet be generalised*. There are two impediments.

Firstly, all of the above data (quantitative and qualitative) relate to the specifics of healthcare management and the preferences of assorted stakeholder groupings in *that* location and *that* time. It remains a unique case study. The second and more interesting deficit stems from a habitual limitation of such qualitative analysis, namely that ‘the analysis of variation between *informant types* is not explicitly reported’ ([32], our italics). Thus, quite typically and as in the summary above, Grimshaw et al. produce a list of assorted ‘themes’ describing the many reasons why practitioners may overlook or resist the bespoke guideline [31]. What is clear from their content, but what is not reported, is that these divergent reactions to the PEM will be particular to specific sub-groups of practices and practitioners. Only some will prefer to trust their own judgement; only some will lack administrative support; only some of their patients will have access problems. And without knowing the exact identity and relative magnitude of these various constituencies, we cannot know whether the same net outcome would follow in other settings. We cannot generalise.

To summarise, adding qualitative description to quantitative appraisal provides a more comprehensive evidence-base to understand the fate of an intervention but additional research strategies are required to judge whether that destiny is likely to be repeated.

### Step two

Marks the beginning of the strategy to better organise these evidential fragments. The key is the introduction of theoretical constructs to widen the explanatory scope of such local findings. This approach eschews the statistical notion that generalisation is based on typicality – i.e., the claim that the intervention studied is ‘representative’ of a larger population of interventions from which it is drawn. The PEMs programme is a complex system consisting of an adaptive intervention, a location, an administrative system, a communication pathway, a patient population, several layers of practitioners, a form of financial regulation, a type of healthcare service, and so on. No study can claim to be representative of all of these features.

The alternative suggested here replaces the ‘programme’ with the ‘programme theory’ as the basic unit of analysis. Programme theories refer to the basic ideas behind an intervention, the reasoning put forward as to why it should work. Such theories are generic. They crop up over and again in programme planning and policy making (for an overview of implementation science theories see Nilsen [33]). The hallmark of such theories is that they operate at a level abstraction beyond that of the concrete interventions such as the one under inspection here. Abstraction, or abduction as it is sometimes called, is itself a powerful ally of generalisation [34]. We explain a particular event as a recognisable case of a wider class of cases, as a variation upon a theme, about which we already have some foreknowledge. Existing understanding delivers provisional ideas on strengths and weakness of that class of programmes, which provide insight on what to expect in any novel application, which insight is then further refined in a closer inspection of each incarnation of the programme theory.

Thus, instead of regarding the ‘2005-Onatario-printed-educational-messages-scheme-to-increase-practitioner-referrals-for-retinal-screening’ as a one-off (which it is), we perceive it as another instance of a well-worn idea (which it also is). So what is PEMS a case of? What is the time-honoured programme theory? I have no access, of course, to the exact thinking of those responsible for this specific intervention. But there are clues aplenty. PEMs are part of the ‘clinical practice guidelines industry’ or ‘standardised care movement’ [35, 36] The generic idea is to bring order and predictability to practitioners’ behaviour by way providing authoritative ‘reminders’, ‘updates’, ‘protocols’, ‘bulletins’, ‘continuing professional education’. In all cases, the message is the medium. The underlying assumption, the common intervention theory, is perfectly simple, namely that – well-informed, well-qualified healthcare practitioners will respond to, and seek to follow, professionally-endorsed, evidence-based information.

The crucial point, to repeat, is that we already know a great deal about this theory and its profound limitations. Information may have august credentials but on its own paper authority is rarely able to countermand deeply-held personal preferences, time pressures, existing routines and institutional constraints. Ironically, the findings from the myriad of studies which told us of the many reasons why practitioners don’t read formal research publications, and which were responsible for ushering in implementation science, recur again in an implementation scheme which tries to engage practitioners with tailor-made, directly-mailed evidential bulletins. We should not have been surprised. Even if there is profound acceptance of the advice on offer, *some* practitioners will not take heed because the daily externalities of their work continue to prevail.

This brings us to the next feature of a programme theory approach. These theories, like the interventions they underpin, are frail and fallible. We thus know a great deal about ‘guideline theory’ because, in scores of applications, it has been tried and found wanting. Thus, in the same way that we abstract the ideas that underpin an intervention, we also need to further build programme theories that provide abstract hypotheses about the conditions that account for success or failure. Policy makers have one set of ideas about a programme, which are often met with the quite different ideas of the practitioners. The hallmark of the programme theory approach is that it assumes mixed outcomes. In the present case, the task is to construct theories about which type of practitioners in which type of practice, are likely to heed (or disregard) the guidance.

Although the aim is to treat them as abstract, formal hypotheses, there is nothing esoteric or erudite about such theories.^1^ For instance, if one refers back to Grimshaw et al’s qualitative sub-study on responses to the retinopathy PEM [31], there are several *implicit* and *untested* hypotheses suggesting which practitioners and which practices might harbour differential responses. For instance, perhaps the simplest of the many themes uncovered for not adhering to the bespoke guidance is ‘confidence in their own clinical assessment’. Which subgroup of practitioners might so reason? An elementary hypothesis is that it is a response which grows with practitioner experience, a proposition which could be then tested empirically with a comparison of change in referral rates between ‘veterans’ and ‘newcomers’.

The real point of treating this ‘experience hypothesis’ as a programme theory is that we have foreknowledge of it. We know from a myriad of studies that guideline adherence is sub-optimal. We thus have a solid expectation that an ‘experience differential’ might crop up as issue in the thousands of other guidelines that exist for every condition, test and treatment. But what we don’t know is the way in which experience makes a difference. Experience is more than the number of years a practitioner spends in harness. Experience also brings with it autonomy, seniority, management responsibilities, professional commitments, specialist knowledge, increasing familiarity with conditions, with patients and with ‘the system’, and so on. There will always be some subtle variations in what constitutes ‘experience’ and by following the idea and its aftermath through a range of case studies we can build up an understanding of its differential impact. Theories are there to be tested and refined, retested and re-refined – and it is out of this process that generalisation occurs.

### Step three

So how should programme theories be tested? A crucial step here is to change emphasis on what constitutes the explanandum in trial research. All KT interventions have mixed fortunes and explaining multiformity should be key objective. The analytic focus should be on ‘outcome patterns’ rather than ‘outcomes’ [37] or on ‘heterogeneous effects’ rather than ‘net effects’ [38]. This proposition has met with considerable resistance. The standard model, even after the advent of pragmatism, is to compare outcomes in treated and untreated groups, the PRCT mustering enough statistical power to reliably detect a net effect as the basis for testing what is still seen as the fundamental outcome question – has the intervention ‘worked’? With increasing frequency, qualitative inquiry is added to build a composite picture of the reasoning of the key recipients, which may have contributed to the observed effect.

This model has persisted despite a considerable *clinical* literature demonstrating that, almost without exception, there are subjects who experience greater and lesser benefits *within* the ‘treated population’ [38]. The common sense notion that treatments don’t work for everyone is captured in clinical terms as heterogeneity of treatment effects (HTE) and its implications are considerable, as in this famous quotation from Kravitz et al.:

‘When HTE is present, the modest benefit ascribed to many treatments can be misleading because modest average effects may reflect a mixture of substantial benefits for some, little benefit for many and harm to a few’ [39].

This proposition has even more currency if we ponder the construction of pragmatic trials. HTE is present even in phase three drug efficacy trials, which have very carefully delimited inclusion and exclusion criteria and which deploy a multitude of further controls [40]. The very idea of pragmatic trials is to investigate in real-world environments in which such controls are loosened. On every dimension of the PRECIS graphic there will be in-built, input variation. It follows that HTE is particularly prominent in PRCTS due to their very design, a hugely profound but largely ignored observation first made by Segal et al. [41]. The significance of the summary result, the net treatment effect, is thereby diminished. In the case of social programmes like PEMS, the average and null treatment effect may well reflect, to paraphrase Kravitz, an information campaign that has had substantial influence on certain practitioners, gone unnoticed by others by many, and acted as another bothersome disincentive to a few.

Such an eventuality tends to be overlooked in PRTs in general and in our particular example of the PEMS trial. The priority of trialists is always to protect internal validity by ensuring, though randomisation, that there is a balance of characteristics and potential predispositions between experimental and control groups. From this perspective, Zwarenstein et al., report a satisfyingly close correspondence between experimental and controlled practices in terms of ‘gender composition’, ‘place of training’, ‘practice size’, and indeed in our highlighted example, ‘years of experience of practitioners’ [17]. The fact that these and other features of the recipients are distributed evenly across experimental and control conditions does not mean that circumstances that they reflect have no bearing on whether educational updates are heeded. It still leaves open the possibility that there are substantial sub-group and sub-processual differences in response to the guidance which, as in Kravitz’s scenario, may remain undetectable in the net effect.

This eventuality can be investigated by identifying and comparing outcomes across differently disposed groups. Qualitative analysis can provide important clues on these likely predispositions. But again the prize of understanding heterogeneous outcomes often fails to eventuate because of a rather different tradition in this form of inquiry. Qualitative analysis has long cherished ‘thick description’ [42]. As in Grimshaw’s sub-study [31], the aim is to capture, often through thematic analysis, the entire range of attitudes towards the programme. The intention of much qualitative analysis is to be comprehensive rather than analytical. The aim is to reach ‘saturation’ in the description of the subjects’ dispositions rather than follow through to the behavioural consequences of the diverse dispositions.

The inevitability of irregular impact in interventions investigated in implementation science changes the research question. The ultimate objective is not to rule on whether interventions work. Rather what needs to be researched, understood, advised upon and exploited is their differential impact.

### Step four

Having speculated upon a revised agenda for implementation science, namely to investigate the manifold contingencies that contribute to the successes and failures of programme theories, it remains to provide an account of the research designs that can accomplish this task. I have already advocated the need for a theory-driven approach involving sequences or series of studies, employing mixed-method approaches. Clearly, there are a range of prospective and retrospective designs that would fit this bill. But what I want to emphasise here are two simple analytic strategies that are key in the search for generalisable evidence.

In the vernacular of case study research, these strategies are referred to as ‘within-case’ and ‘cross-case’ analysis [32, 43]. How might these be deployed in the investigation of guideline theory? In the former, different sub-groups of the recipients of a guideline are identified, differences in their predispositions towards and resultant behaviour in respect of the advice are hypothesised, and data is generated to test these conjectures. Achieving guideline adherence, however, reflects not only on the people involved but their roles, their networks, their organisations and the wider regulatory environments in which they work [44]. To tease out these influences requires cross-case study, with comparisons chosen to reflect outcomes that may be generated in different institutional locations in receipt of the guideline.

Learning increases as these analytic cuts are applied sequentially. Existing knowledge of the strengths and the weaknesses, the winners and the losers, of previous incarnations of the programme theory are built into the choice of comparators in investigation 1. Some of these hypotheses will be supported and some will be refuted. This provides the spur to investigation 2, which revises the programme theory and adapts the comparison groups in an attempt to explain the emerging heterogeneity of outcomes. Revised programme theories remain fragile. They meet with conformities and anomalies, and the research continues through more case studies in an attempt to iron them out (recall Fig. 2).

This completes a blueprint of a mixed-method strategy for evaluating generic implementation strategies. Rather like clinical guidelines, methodological precepts only have significance if they find practical use. This brings me to the final entreaty in a paper of entreaties, namely to welcome the growing usage of within-case and cross-case analysis in implementation science. I only have space here to sketch four brief examples that exemplify the case for multi-site, mixed-method, theory-driven case studies. These illustrations take off where the previous examples have left off, namely with our initial and highly fallible programme theory that healthcare practitioners will respond to professionally-endorsed, evidence-based guidelines and our first-guess, common-sense implementation theory that ‘practitioner experience’ will make a difference in their uptake.

Gove et al. [44] examine responses of orthopaedic surgeons to NICE guidelines on total hip replacement in three NHS hospitals. These senior clinicians responded to the guidance in quite different ways according to setting. In case A, an academic centre located within a trauma and orthopaedic unit, surgeons held a ‘positive view of formal, codified knowledge’, were ‘accustomed to answering questions using a larger population frame of reference’ and complied their ‘own protocol documentation linked to a piece of clinical guidance’. Case C, an orthopaedic department in a teaching hospital, provided the extreme contrast, with surgeons reporting that they ‘had never seen their organisations NICE process’, which ‘belonged to the managerial and administrative domain’. Here, surgeons preferred ‘resilient, experiential knowledge built up over time’ based on ‘the innate feel of surgery’. Experience drives these senior clinicians in opposite directions – according to context.

Rycroft-Malone et al. [36] examined responses to ‘protocol-based care’ in a multi-case study across nursing, midwifery and health-visiting, etc. The tools met with quite different responses. In line with our primitive hypothesis, the authors report that those practitioners with more experience ‘either did not refer to them or used them flexibly’. Also, as per thesis, junior practitioners perceived the protocols as ‘useful information resources’. But there are a couple of significant twists. In some contexts the authority carried by guidelines is seen as empowering. The standardisation of what constitutes good practice, ‘enabled the extension of traditional roles and facilitated autonomous practice, which in turn resulted in more nurse and midwifery led care and services’. By contrast, ‘in contexts in which there are frequent staff changes or which relied on agency staff’, the guidance was ‘included in induction materials and competency assessments’. In these situations relatively high levels of adherence follows from the guideline being conscripted and coached.

Moule et al. [45] evaluated a quality improvement programme incorporating NICE guidelines on using anticoagulants to reduce atrial fibrillation (AF) strokes across six general practices in the UK. The resources were used variably. Again GP’s experience and preconceived ideas proved important – but with further variations on the theme. In this instance, some of the most experienced practitioners with personal expertise took leadership of the scheme and helped propel guidance into a review and follow up scheme. Other practices, often where the GP worked in isolation, lacked the ‘system “mind-set” ... to enable them to track/ monitor patients’. In these cases the reliance on experience and personal judgement was in large part due to a lack of formal infrastructure to service the guidelines.

Spyridonidis and Calnan [46] used a longitudinal, within-case and cross-case comparison to evaluate how the implementation of two NICE guidelines, on chronic heart failure (CHF) and obesity, unfolded over time. Their emphasis is on ‘whole system’ adaptation to the guidelines and so they examine the perspectives of professional executives, senior and middle managers, hospital clinicians, GPs, nurses and allied health professionals. The guidelines, as ever, are followed ‘variably’; their implementation juddering ‘back and forth’ as different stakeholders ponder their rival consequences. For instance, one option on the NICE guideline on obesity was for bariatric surgery. Following an upsurge in bariatric referrals, management rapidly rewrote the guideline to ration an expensive procedure. In another example, the NICE guideline on CHF recommended use of beta-blockers, which were not included in a further NHS scheme, the Quality and Outcomes Framework (QOF), which remunerated GPs for meeting a range of performance indicators. Implementation of the NICE recommendation stalled awaiting convergence between the respective policies.

Many, many more such case studies could, of course, be consulted to deepen an understanding of the erratic uptake of the guidelines.^2^ The point is that by starting with a relative abstract programme theory, rather than a forest of separate initiatives, a research programme of within-case and between-case studies can begin to unlock the variations in outcomes that routinely follow on guideline implementation. Thus in the present case, I began with the crude implementation theory that junior staff were more inclined to respect professionally-endorsed guidance whereas experienced practitioners were more likely to trust their own judgement. This theory is full of holes or, as Popper might prefer, is in urgent need of ‘error elimination’. Thus, we discover experienced practitioners can be enthusiastic exponents of guidelines – if they work in institutions oriented to wider epidemiological perspectives. We discover that some veteran practitioners’ preference for their own judgement really resides in their lack the system infrastructure to implement a new guideline. We discover that some relatively junior staff do indeed embrace guidelines – but do so on the basis that they offer autonomy and empowerment, rather than delivering research wisdom. We discover that other subordinate groups follow research-endorsed guidelines simply because they are embedded in induction and training. We discover that practitioners well-disposed to new guidelines are often thwarted in applying the schemes because other stakeholders (often with more power) perceive the changes will have damaging consequences in their operational spheres.

## Conclusions

There are clinical practice guidelines (sometimes multiple) for every conceivable condition and its associated treatments. Healthcare policy-makers are inclined to be over-impressed by the uniqueness and originality of their brainchildren. They want their efforts evaluated and research teams are ever willing to pocket funding to tackle each schema on the basis that it is ‘novel’. This has resulted in a research progeny of hundreds of separate inquiries which, almost without exception, show that guideline uptake is mixed across its different individual and institutional recipients. The danger in this atomised approach is that implementation science agglomerates rather than accumulates. There is an underlying pattern to the divergent responses to such KT schemes but this is missed if the inquiry tries to wash out sub-group difference through randomisation or simply describes an amalgam of local reasons why local practitioners fall short of compliance.

The core petition in this paper is to resist the temptation to begin each inquiry from scratch. To be sure, each and every clinical guideline is unique and aimed at a unique body of practitioners. But there are a limited number of ways in which one group of practitioners can influence the behaviour of another group of practitioners and every reason to suspect there will be commonalities in the response. Such recurring patterns can be unearthed if research begins with programme theories and sets in place, prospectively or retrospectively, a series of case studies with which to test and refine those theories. Evidence accumulates by building a picture of the conditions and caveats which surround each programme theory and these qualifications form the basis of advice to future practitioners. Rather than offering exhortations to do this or do that (advice which, as we have seen, is generally scattered to the wind) knowledge transfer schemes should spell out the alternative choices and the multiple consequences associated with a family of programmes (in order to help practitioners think through the potential and pitfalls of their own schemes).

## Data Availability

N/A

## Abbreviations

AF: Atrial fibrillation
CHF: Chronic heart failure
HTE: Heterogeneity of treatment effects
KT: Knowledge transfer
NHS: UK National Health Service
NICE: The National Institute for Health and Care Excellence, UK
PCRCT: Pragmatic cluster randomised controlled trial
PEMS: Printed educational messages
PRCT: Pragmatic randomised controlled trial
PRECIS: PRagmatic Explanatory Continuum Indicator Summary
QOF: Quality and Outcomes Framework
RCT: Randomised controlled trail

## Footnotes

1 It will be recalled that the PEMS sub-study [31] labels itself as a ‘theory-based process evaluation’. At the risk of complicating matters, some difference between the authors’ strategy and the approach advocated in this paper are worthy of note. Grimshaw et al. utilise the ‘theory of planned behaviour’ (TPB) to explain the paradoxical outcomes of the main trial. TPB is an example of what Nilsen [32] refers to as ‘classical theory’, that is to say a theory that originate from fields *external* to an intervention under study. In this case TPB is a generic psychological theory on decision-making, which explains why peoples’ best intentions do not always lead to action. Put simply, the theory is that the motivated individual will only act on their intentions *if* they have belief in their own ability to perform the requisite actions. It turns out many practitioners in PEMS study were positively inclined to increase retinopathy referrals *and* confident in their ability to do so – and yet still do not change their behaviour. Interesting and carefully researched as this finding is, it is arguably a refutation of TPB, rather than an exemplification. A standard charge against this psychological theory is that it cannot account for, as in this instance, environmental and organisational aspects of decision making. In general terms, my preference is to start with more mundane programme theories, which more closely reflect the thinking of planners and practitioners, and which render these stakeholders more likely to act on any research which probes into them.

2 Note that the claim here is *not* that case study design is inherently superior to any other research design. The four examples here all have their methodological flaws and limitations. One important instance is that all four rely on *self-report* on the matter of whether practitioners make use the guideline. In this respect PRCTs, like [28], are superior because they test whether the guideline produce changed *behaviour*. Individual case studies are always open to improvement by optimising the balance of quantitative and qualitative methods. But collectively they are paramount because they provide multiple tests of the same programme theory.
